# Identification and expression analysis of expansin gene family in *Salvia miltiorrhiza*

**DOI:** 10.1186/s13020-023-00867-w

**Published:** 2024-02-04

**Authors:** Yunyun Li, Bin Li, Qiyue Pang, Yaoyu Lou, Donghao Wang, Zhezhi Wang

**Affiliations:** 1 Key Laboratory of the Ministry of Education for Medicinal Resources and Natural Pharmaceutical Chemistry, National Engineering Laboratory for Resource Development of Endangered Crude Drugs in Northwest of China, Shaanxi Normal University, Xi’an, 710062 China; 2 Xi’an Botanical Garden of Shaanxi Province (Institute of Botany of Shaanxi Province), Xi’an, China

**Keywords:** *Salvia miltiorrhiza*, Expansin, Bioinformatics, ABA, Abiotic stress, Subcellular localization

## Abstract

**Background:**

Expansins (EXP) are important enzymes that are involved in the extension of plant cells and regulation of root configurations, which play important roles in resisting various stresses. As a model medicinal plant, *Salvia miltiorrhiza* is well recognized for treating coronary heart disease, myocardial infection, and other cardiovascular and cerebrovascular diseases; however, the *SmEXP* gene family has not yet been analyzed.

**Methods:**

The *SmEXP* family was systematically analyzed using bioinformatics. Quantitative real-time PCR was employed to analyze the tissue expression patterns of the *SmEXP* family, as well as its expression under abscisic acid (ABA) treatment and abiotic stress. Subcellular localization assay revealed the localization of *SmEXLA1*, *SmEXLB1*, and *SmEXPA2*.

**Results:**

This study identified 29 SmEXP that belonged to four different subfamilies. *SmEXP* promoter analysis suggested that it may be involved in the growth, development, and stress adaptation of *S. miltiorrhiza*. An analysis of the expression patterns of *SmEXP* revealed that ABA, Cu^2+^, and NaCl had regulatory effects on its expression. A subcellular localization assay showed that SmEXLA1 and SmEXLB1 were located on the nucleus and cell membrane, while SmEXPA2 was located on the cell wall.

**Conclusion:**

For this study, the *SmEXP* family was systematically analyzed for the first time, which lays a foundation for further elucidating its physiological and biological functionality.

**Supplementary Information:**

The online version contains supplementary material available at 10.1186/s13020-023-00867-w.

## Introduction

*Salvia miltiorrhiza* Bunge (*S. miltiorrhiza*) is a well-recognized model medicinal plant, the dried roots of which are used for the treatment of cardiovascular and cerebrovascular diseases [1–4]. Plant cell walls are dynamic and complex structures that determine the shapes and sizes of plant cells by controlling the degree and direction of cell elongation [5]. Through a combination of cell wall loosening and cell expansion, plant cells have the capacity grow to more than 100 times the size of their initial meristem [6].

EXPANSIN (EXP) is a key regulator of cell wall elongation during plant growth [7, 8], which can be divided into four subfamilies: EXPA (Expansin A), EXPB (Expansin B), EXLA (Expansin-like A), and EXLB (Expansin-like B) [9]. EXP are a multi-gene family that are widely distributed in angiosperms, gymnosperms, ferns, and bryophytes. To date, the genome-wide identification and functional verification of EXP has been conducted for many plants, including *Arabidopsis thaliana*, *Oryza sativa* [10], *Nicotiana tabacum* [11], *Solanum lycopersicum* [12], *Populus* [13], *Triticum aestivum* [14], *Glycine max* [15], and *Malus domestica* [16].

EXP was initially discovered in *Cucumis sativus* in 1992. EXP can induce heat deactivated cell walls to stretch again; however, the stretching process is influenced by pH with the highest activity of this expansive protein occurring in the range of from pH 4.5–6. For example, AtEXPA1 requires low pH activation to exert the relaxation of cell wall activities [17]. There is ample evidence that EXP can relax and stretch the cell wall, albeit the mechanism is not clear. Currently, there are two hypotheses regarding the kinetics of EXP, with one being the acid growth theory and the other, the mechanistic non-enzymatic action model [10, 18].

The primary function of EXP is to cleave the non-covalent bonds between cellulose and other polysaccharides in the cell wall, such that it becomes relaxed and increasingly flexible to regulate plant growth and development [19, 20]. In addition to relaxing cellular walls, EXP also plays an important role in seed germination [21], leaf and stem development [22, 23], root elongation [24], fruit ripening [25], and plant stress resistance [26, 27].

It was found that the overexpression of *NtEXPA1* in tobacco resulted in increased leaf and stem sizes [28]. OsEXPB2 affects root structures and plant heights by inhibiting cell growth [29]. Interference with *OsEXPA8* in rice results in shorter primary fibrous roots and fewer lateral roots [30]. Other root hair EXPAs (*OsEXPA30* and *AtEXPA7*) can complement the *OsEXPA17* mutant to restore root hair elongation [31]. GmEXPB2 can adapt soybean to low phosphorus stress by modifying lateral root development and it configurations [32]. *HvEXPB7* has also been shown to be associated with root hair formation in *Hordeum vulgare*, which was significantly inhibited after *HvEXPB7* silencing [33]. Another study showed that *SlEXP1* expression was closely related to the degree of fruit softening during fruit ripening [34]. ZmEXPB15, ZmNAC11, and ZmNAC29 impact the early development of corn kernels and regulates the grain size and weight by promoting the elimination of nucellar tissue [35]. Further, GhEXPA3-1 promotes fibrocyte elongation through the brassinolide signaling pathway [36].

Studies have revealed that EXP plays an important role in plant responses to abiotic stress. Compared with wild-type tobacco, *NtEXPA11* overexpressed strains had strong roots, significantly increased leaf and internode length, greater cell wall flexibility, and improved tolerance to drought and salt stress [37]. The overexpression of *EXPA4* in tobacco confers greater tolerance to salt and drought stress, which results in less cell damage and a higher fresh weight [38]. The overexpression of *TaEXPA2* increased drought stress tolerance in transgenic tobacco [39] as well as wheat [40]. CqEXPA50 plays an important role in the exposure of quinoa seedlings to salt stress [41]. The overexpression of *AtEXP3* and *AtEXPB1* can enhance the sensitivity of Arabidopsis to salt stress [42]. OsEXPA7 enhances salt stress tolerance by coordinating sodium transport, ROS clearance, and cell wall loosening [43]. Further, plant cell walls can prevent heavy metals from entering its interior [44, 45].

Multiple studies have indicated that EXP can affect almost every plant growth stage. Meanwhile, *S. miltiorrhiza* is affected by several abiotic stress factors such as salt, drought, and heavy metals in the soil during its growth process. As the first barrier against external environmental stress, cell walls play a critical role in cell responses to external stress. Thus, it is essential to identify and analyze SmEXP to establish a foundation for the further study of its functionality.

## Materials and methods

### Identification and characterization of SmEXPs

The AtEXPs sequence was obtained from the TAIR database (https://www.arabidopsis.org/) [46], while the OsEXPs sequence was acquired through the Rice Genome Annotation Project (http:/ /rice.plantbiology.msu.edu/). The *S. miltiorrhiza* genome file was obtained from the China Traditional Chinese Medicine Data Center (https://ngdc.cncb.ac.cn/). BioEdit software (Borland, Scotts Valley, CA, USA) was used to compare AtEXPs with the *S. miltiorrhiza* protein database to obtain candidate proteins. The candidate SmEXPs protein sequences were submitted to InterProScan (https://www.ebi.ac.uk/interpro/), and a CD search (https://www.ncbi.nlm.nih.gov/cdd/) was conducted to further confirm whether Pollen_allerg_1 (PF01357) and DPBB_1 (PF03330) domains existed in the retrieved candidate SmEXPs protein sequences. It was confirmed that there were 29 SmEXPs in *S. miltiorrhiza*.

### Phylogenetic analysis and *cis*-elements analysis

The phylogenetic tree of AtEXPs, OsEXPs, and SmEXPs proteins was constructed using MEGA X [47], which was beautified with the online website Evolview (http://www.evolgenius.info/evolview/).

Promoter sequences of 29 *SmEXPs* (− 1000 bp) were used to predict the *cis*-elements of the SmEXPs promoter region using PlantCARE (http://bioinformatics.psb.ugent.be/webtools/plantcare/htmL/) [48], whereas Tbtools v1.089 software (Chengjie Chen et al., China) [49] was used for visualization.

### Physicochemical properties, gene structure, and Ka/Ks analysis

The SmEXP sequences were submitted to the ProtParam tool website (https://web.expasy.org/protparam/) [50] for an analysis of their amino acid numbers (AA), molecular weights (Mw), protein instability indices, isoelectric points (pH), and grand average of hydropathicity (GRAVY) and aliphatic indices. Plant-mPLoc (http://www.csbio.sjtu.edu.cn/bioinf/plant-multi/) was used for subcellular localization analysis. The gene structures and motifs of the *SmEXPs* were studied by Tbtools, and the Ka and Ks values were calculated using TBtools.

### GO analysis and prediction of protein interactions

GO and KEGG analysis was performed using OmicShare tools, a free online platform for data analysis (https://www.omicshare.com/tools). The Orthovenn2 website (https://orthovenn2.bioinfotoolkits.net/home) was employed to search for the homologous *EXP* genes of *A. thaliana* and *S. miltiorrhiza*, while the sequence consistencies of AtEXP and SmEXP were calculated using DNAMAN (LynnonBiosoft, USA). The homologous genes of *Arabidopsis* that corresponded to *SmEXPs* were uploaded to the String10 website (http://string-db.org/) to visualize the interaction network.

### Plant materials and treatments

*Salvia miltiorrhiza* plants were grown in a field nursery. The four tissue components of two-year-old *S. miltiorrhiza* plants (roots, stems, leavess, and flowers) were obtained from a resource nursery. The 2-month-old *S. miltiorrhiza* tissue culture seedlings were employed for hormone and stress treatments. The 2-month-old *S. miltiorrhiza* plants were sprayed with 100 μM ABA, 100 mm NaCl, and 200 μM Cu^2+^, respectively, and then sampled according to selected time points.

### RNA extraction and quantitative real-time PCR (qRT-PCR) analysis

For qRT-PCR analysis, the total RNA was extracted from the *S. miltiorrhiza* plants using Plant Total RNA isolation kits (Aidlab, Beijing, China). Reverse transcriptase was employed to reverse transcribe the whole RNA to cDNA (Invitrogen, Waltham, MA, USA).

All qPCR assays were performed with the CFX96 real-time system (Bio-Rad, USA) using *S. miltiorrhiza* β-actin (DQ243702) [51] as the internal control, and quantification was performed by SYBR (Takara, Japan). Each reaction had three biological and technical replicates, using 40-fold diluted cDNA as a template. The expression of *SmEXPs* was calculated via the comparative CT method (2^−ΔΔCT^) [52]. One-way ANOVA was employed to examine the statistical significance of the data. The primer sequences used in this study are listed in Additional file 1: Table S1.

### Subcellular localization of *SmEXPA2*, *SmEXLA1*, and *SmEXLB1*

The *SmEXPA2*, *SmEXLA1*, and *SmEXLB1* without stop codons were cloned into the pEarleyGate103 (35-GFP) vector to obtain the recombinant vector GFP-SmEXPA2/SmEXLA1/SmEXLB1. The recombinant vector GFP-SmEXPA2/SmEXLA1/SmEXLB1 and 35-GFP were transformed to Agrobacterium EHA105. The bacterial solution carrying GFP-SmEXPA2/SmEXLA1/SmEXLB1 and 35-GFP were impregnated with onion epidermis and cultured for 2 days, respectively. After soaking, the onion skin was cultured in the dark on MS medium for 2 days, and then observed with a fluorescence microscope (Leica DM6000B, Wetzlar, Germany).

## Result

### Identification of SmEXP family

A total of 29 SmEXPs in the *S. miltiorrhiza* genome were found to contain both Pollen_allerg_1(PF01357) and DPBB_1(PF03330) domains. The sequences of each SmEXPs are shown in Additional file 2. The physicochemical properties of SmEXPs were then further analyzed (Table 1). The number of amino acids encoded by SmEXPs ranged from 241 to 328 aa, whereas the molecular weights of the proteins ranged from 26.05 to 36.19 kDa. The SmEXPs had a pI that ranged from between 4.83 and 9.64.

**Table 1:** Physicochemical properties of SmEXPs. Nomenclature, peptide lengths, molecular weights (MW), theoretical isoelectric points (pI), instability indices, aliphatic indices, Grand Average of Hydropathicity (gravy)

Gene name	AA	Mw(kDa)	pI	Instability index	Aliphatic index	GRAVY
SmEXPA1	247	26.57	7.54	31.96	71.94	− 0.011
SmEXPA2	253	26.99	8.96	32.57	70.28	− 0.011
SmEXPA3	245	26.37	9.57	36.58	62.94	− 0.214
SmEXPA4	247	26.64	8.66	28.37	70.73	− 0.134
SmEXPA5	255	27.12	8.07	33.17	66.27	− 0.196
SmEXPA6	248	27.01	9.63	39.47	72.74	− 0.019
SmEXPA7	248	26.24	9.53	39.43	69.72	− 0.077
SmEXPA8	247	26.05	9.25	37.59	62.87	− 0.106
SmEXPA9	263	28.22	9.3	29.92	71.33	− 0.003
SmEXPA10	261	28.00	9.64	29.33	72.95	− 0.012
SmEXPA11	261	28.09	9.59	33.07	72.22	− 0.025
SmEXPA12	256	27.87	9.46	33.92	65.98	− 0.082
SmEXPA13	251	26.74	9.21	43.61	60.32	− 0.11
SmEXPA14	255	27.65	8.95	30.02	60.82	− 0.177
SmEXPA15	256	27.82	9.33	30.86	61.02	− 0.141
SmEXPA16	264	28.47	8.77	37.54	81.36	0.013
SmEXPA17	250	27.32	8.16	36.39	74.2	− 0.104
SmEXPA18	260	28.26	6.79	30.72	70.08	− 0.072
SmEXPA19	265	28.44	9.05	21.73	65.92	0.026
SmEXPA20	252	27.43	9.21	23.88	63.17	− 0.015
SmEXPA21	328	36.19	6.45	37.15	53.29	− 0.787
SmEXLA1	263	28.47	8.47	41.54	82.81	− 0.059
SmEXLB1	256	28.03	6.29	26.26	81.13	− 0.075
SmEXLB2	257	27.87	4.83	36.61	72.92	− 0.184
SmEXPB1	253	27.08	8.45	38.69	75.97	− 0.051
SmEXPB2	250	26.52	7.45	34.8	79.28	0.088
SmEXPB3	262	28.81	5.42	42.17	70.34	− 0.307
SmEXPB4	267	28.33	5.25	43.2	65.84	− 0.153
SmEXPB5	241	26.12	8.89	43.89	72.86	− 0.188

Furthermore, the GRAVY of SmEXPs was predicted to determine the hydrophilic and hydrophobic properties of the SmEXPs. The analysis revealed that 26 SmEXPs belonged to hydrophilic proteins, and three SmEXPs were hydrophobic proteins. SmEXPA21 exhibited the strongest hydrophilicity, while SmEXPB2 showed the greatest hydrophobicity. There were 24 SmEXPs with a low Instability index (Instability index < 40), indicating that most SmEXPs were stable, which is necessary for their functionality and the maintenance of normal plant growth. Subcellular localization prediction showed that all SmEXPs were localized to the cell wall.

### Phylogenetic and Ka/Ks analyses

**Fig. 1:**
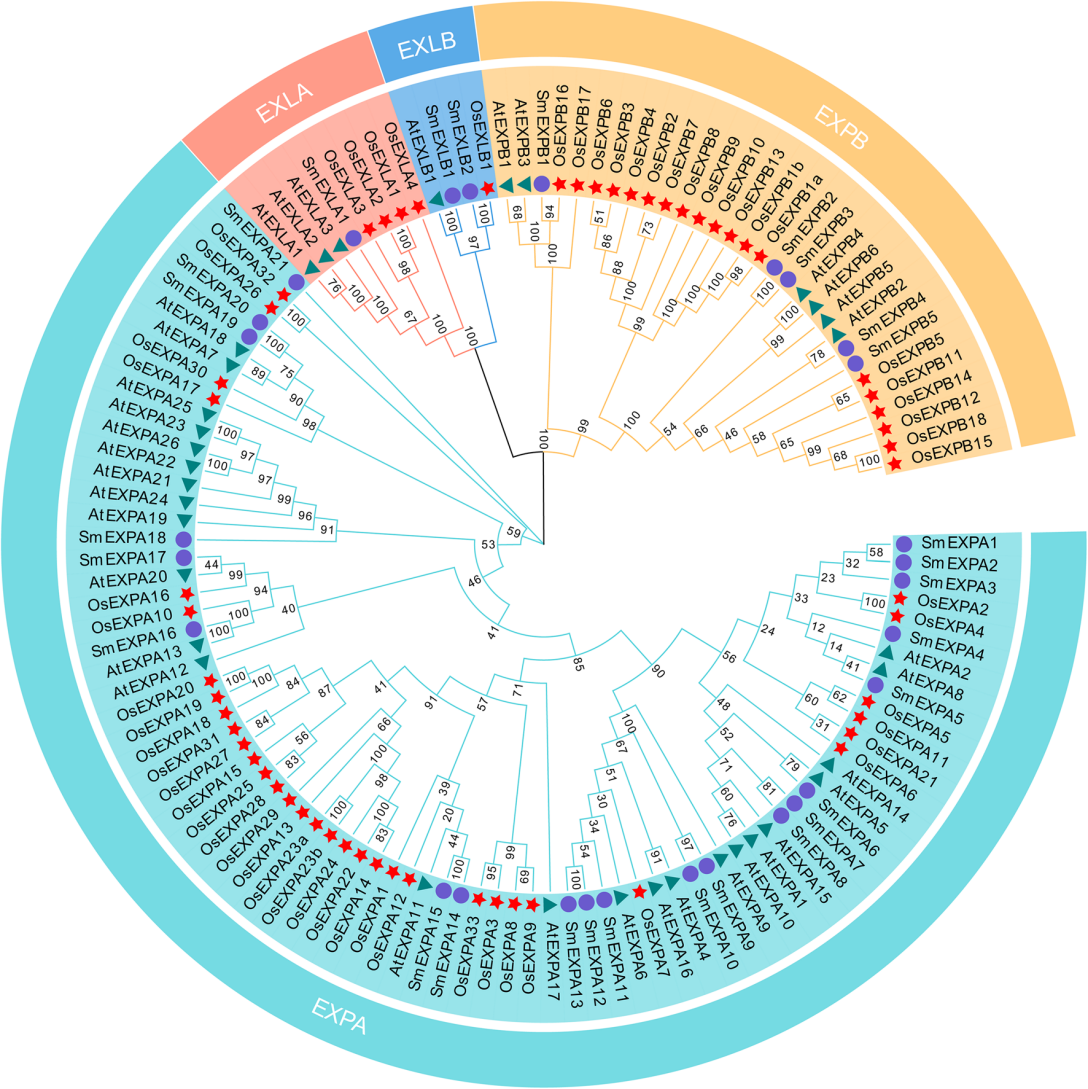
Phylogenetic relationships between EXP sequences. The full-length amino acid sequences of SmEXP, AtEXP, and OsEXP were used to construct the phylogenetic tree using MEGA X via the neighbor-joining (NJ) method. The value at the branch is the confidence level determined by performing 1000 bootstrap tests

To explore the phylogenetic relationships between the SmEXPs, AtEXPs, and OsEXPs, 136 protein sequences were compared and phylogenetic trees were constructed (Fig. 1). The results revealed that the EXP members of *S. miltiorrhiza*, *A. thaliana*, and *O. sativa* could be divided into EXPA, EXPB, EXLA, and EXLB subfamilies. The SmEXPA subfamily, with the highest number, included 21 members. Next, the SmEXPB subfamily had five members, the SmEXLA subfamily had one member, and the SmEXLB subfamily had two members.

According to the sequence similarity of the SmEXP family, 11 homologous gene pairs were selected for prediction (Table 2). It was predicted that the Ka/Ks ratio of all gene pairs were less than 1. This indicated a strong purifying selection in the evolution of the SmEXP family that tended to be more stable, which was conducive to maintaining the conservation of gene family functionality. The Ka/Ks ratio of the SmEXPA12&SmEXPA13 group was highest, which indicated that the evolution rate of this group was faster. Conversely, the Ka/Ks ratio of the SmEXPA6&SmEXPA7 group was lowest, which implied that the amino acid sites in this group were more conserved and not easily changed.

**Table 2:** Ka/Ks values of homologous SmEXP gene pairs

Seq_1	Seq_2	Ka	Ks	Ka/Ks	Purify selection
SmEXPA1	SmEXPA2	0.120933521	0.971354874	0.124499834	Yes
SmEXPA1	SmEXPA3	0.137213793	1.49460299	0.091806181	Yes
SmEXPA2	SmEXPA3	0.14271441	1.63256906	0.087417074	Yes
SmEXPA6	SmEXPA7	0.136601905	2.091241878	0.065320949	Yes
SmEXPA6	SmEXPA8	0.182700541	2.074914015	0.088052102	Yes
SmEXPA7	SmEXPA8	0.087005787	0.74967307	0.116058306	Yes
SmEXPA9	SmEXPA10	0.097360801	0.490746045	0.198393451	Yes
SmEXPA12	SmEXPA13	0.055819961	0.088892722	0.627947487	Yes
SmEXPA14	SmEXPA15	0.137684079	1.800801653	0.076457104	Yes
SmEXPA19	SmEXPA20	0.148863933	0.841209287	0.176964205	Yes
SmEXPB2	SmEXPB3	0.348582239	0.966533627	0.360651952	Yes

### Motif and gene structure analyses

**Fig. 2:**
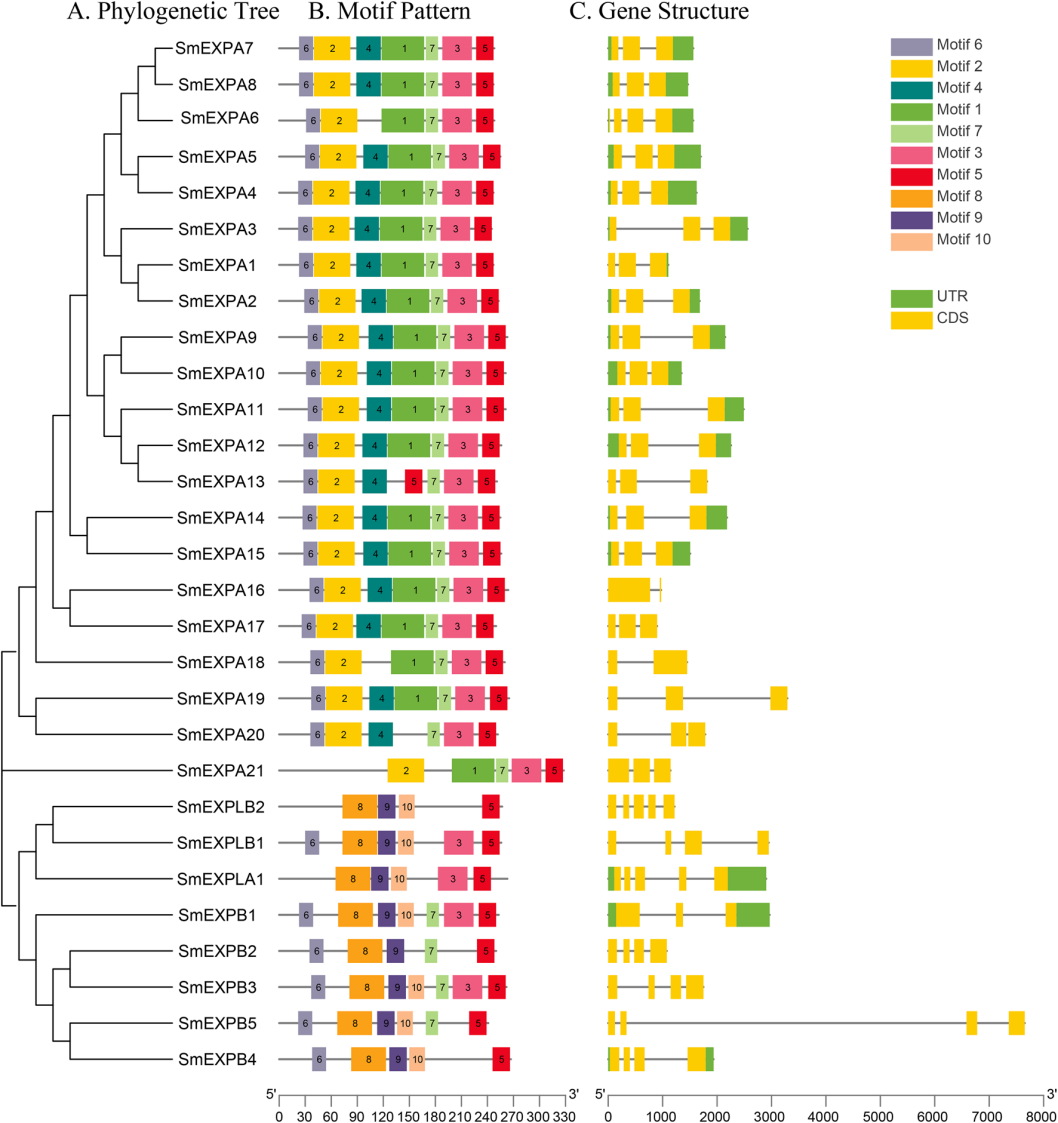
Evolutionary tree, conserved motifs, and gene structures of SmEXP family. **A** Phylogenetic relationship between SmEXPs. **B** Conserved motifs of SmEXPs. **C** Exon–intron structure of *SmEXP*. Yellow rectangles refer to UTR, and green rectangles refer to CDS

The gene structures and conserved motifs within each subfamily were almost consistent (Fig. 2A). Motif 5 was present in all subfamilies, while motif 6 was present in all SmEXPA subfamilies (except SmEXPA21). Motif 7 was present in all SmEXPB proteins (except for SmEXPB4) (Fig. 2B). The motif analysis revealed that the proteins translated from genes in the same subfamily had similar amino acid structures, which may have been related to tandem repetition, random repetition, and insertion during the genome evolution process. Most *SmEXPs* contained one-four introns, *SmEXPA* tended to have two introns, and *SmEXPB* tended to have three introns (Fig. 2C).

### *Cis*-element *SmEXP* analysis

**Fig. 3:**
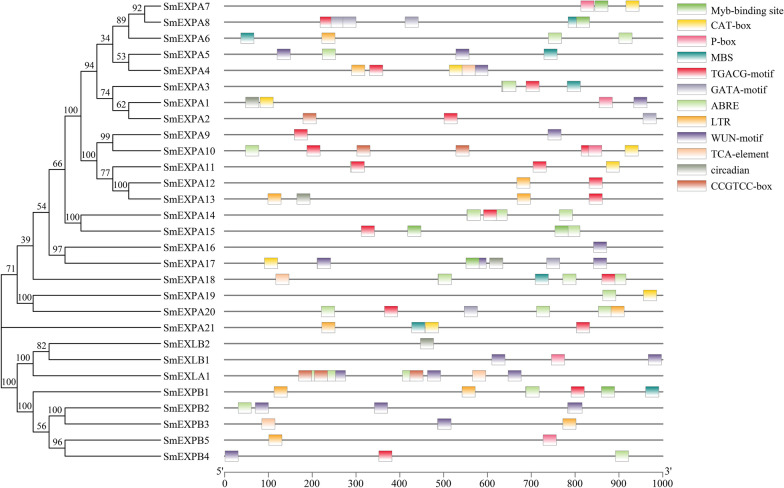
Predicted *cis*-elements in the *SmEXPs* prompters (− 1000 bp). Different *cis*-elements are represented by different colors

A total of 12 *cis*-elements were predicted, while the GATA-motif was associated with light response. The ABRE, TGACG-motif, P-box, and TCA-element were associated with hormone responses, and the WUN-motif was associated with wound response. Circadian was associated with circadian control, whereas CAT-box and CCGTCC-box were associated with tissue-specific and developmental associations, whereas MBS, Myb-binding sites, and LTR were correlated with abiotic stress (Fig. 3). The analysis results suggested that *SmEXPs* may have played important roles in the photoresponse, hormonal response, abiotic stress response-related, tissue-specific, and developmental responses.

### GO annotation and prediction of protein interactions

**Fig. 4:**
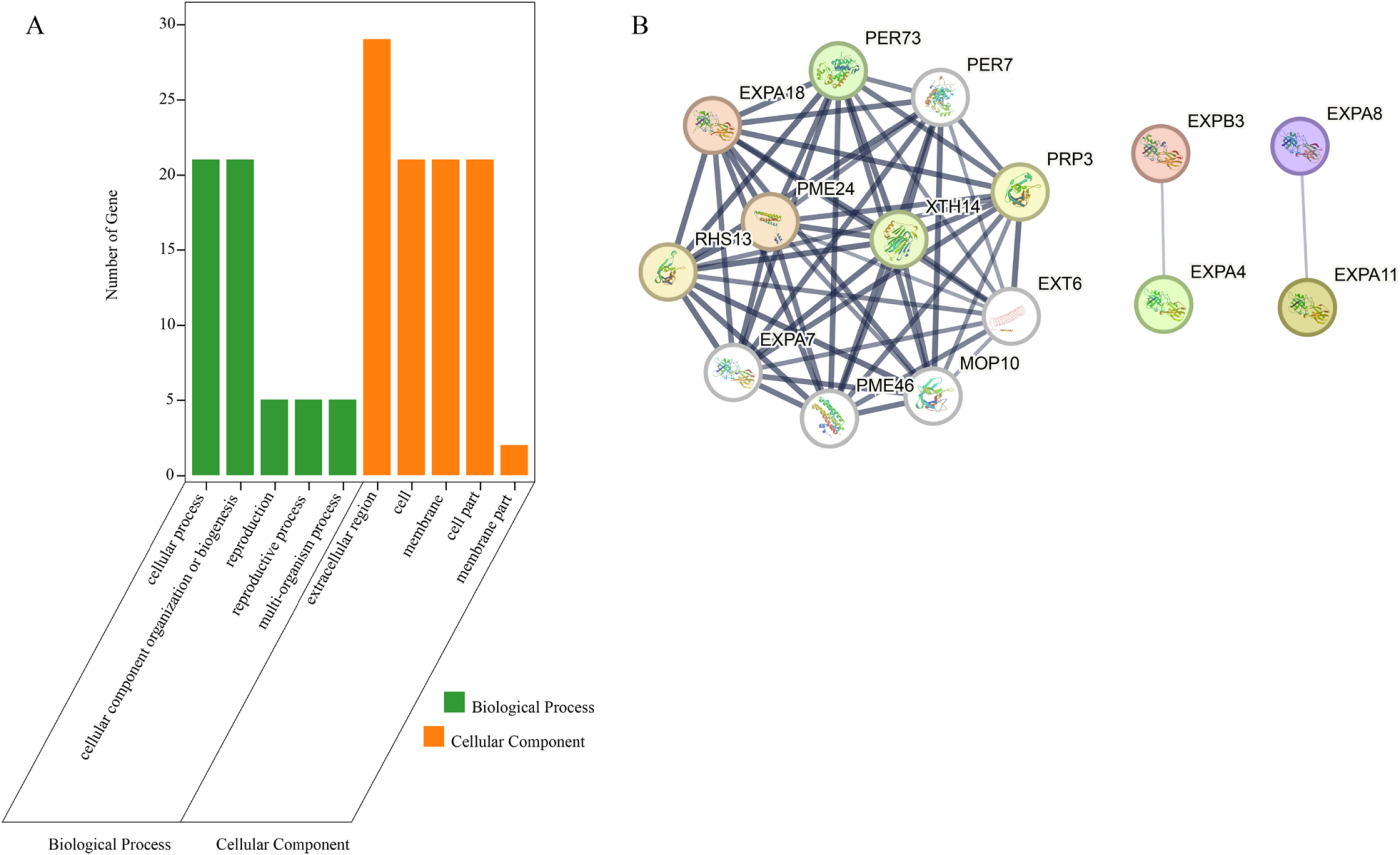
Functional analysis. **A** GO terms enriched with SmEXPs. **B** Protein–protein interaction networks of SmEXPs. The strength of the protein interaction is shown by the line's thickness

The GO annotation was divided into three levels (molecular function (MF), biological process (BP), and cellular component (CC)). The SmEXPs had only biological process and cellular component annotations and GO annotation assisted with understanding the functions of proteins at the molecular level. At the CC level, the SmEXPs were primarily enriched in the extracellular region, while at the BP level the SmEXPs were primarily enriched in cellular processes, cellular component organization, or biogenesis (Fig. 4A).

Orthologous genes often have similar functions [53]; thus, the *Arabidopsis* interaction network to was used to map the potential interactive SmEXP network. OrthoVenn2 was employed to screen the orthologous genes of *SmEXP* in the *Arabidopsis* database (Table 3). The sequence alignment results of orthologous genes are shown in Additional file 3: Fig. S1. The results indicated that the sequence consistency of these 13 pairs of orthologous genes was > 50%. The results showed that AtEXPA4 could interact with AtEXPB3, and AtEXPA8 could interact with AtEXPA11. The interactive network of AtEXP18 was rich, and AtEXP18 could interact with another 10 genes to facilitate its functions (Fig. 4B).

**Table 3:** Orthologous *SmEXP* and *AtEXP* genes

SmEXP Name	AtEXP Name	AtGene ID	Sequence identity
SmEXPA1	AtEXPA8	AT2G40610	75.10%
SmEXPA14	AtEXPA11	AT1G20190	69.80%
SmEXPA19	AtEXPA18	AT1G62980	60.38%
SmEXPB4	AtEXPB2	AT1G65680	53.09
SmEXPA7	AtEXPA15	AT2G03090	81.03%
SmEXPA12	AtEXPA6	AT2G28950	80.54%
SmEXPA11	AtEXPA4	AT2G39700	79.69%
SmEXPA16	AtEXPA13	AT3G03220	71.91%
SmEXLB1	AtEXLB1	AT4G17030	57.75%
SmEXPB1	AtEXPB3	AT4G28250	51.52%
SmEXPA17	AtEXPA20	AT4G38210	54.47%
SmEXLA1	AtEXLA2	AT4G38400	62.41%
SmEXPA18	AtEXPA23	AT5G39280	50.36%

### Analysis of *SmEXP* tissue expression patterns

**Fig. 5:**
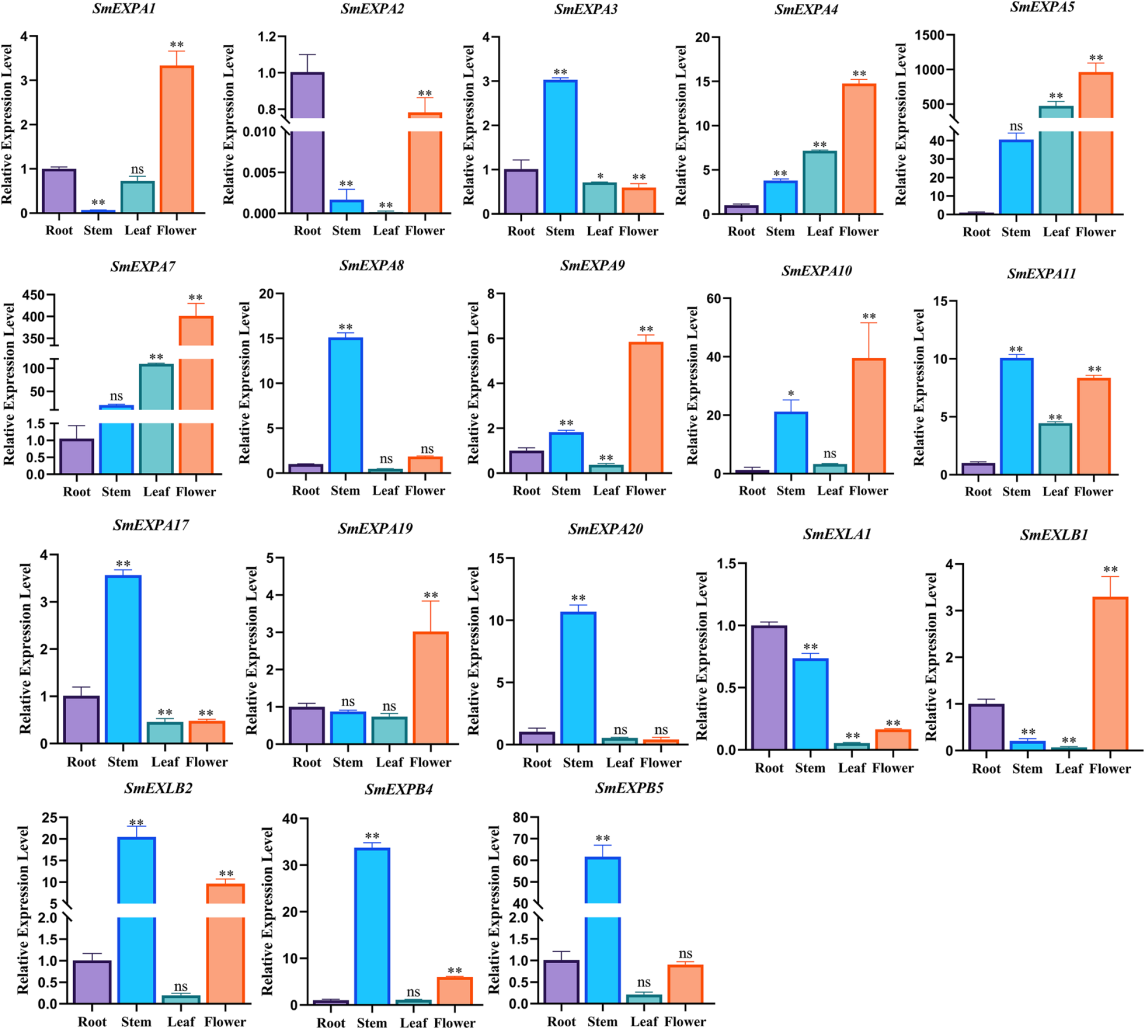
Expression levels of *SmEXP* in roots, stems, leaves, and flowers. The transcript levels were analyzed using the qRT-PCR. Data are shown as means ± SD, n = 3. Asterisks indicate significance differences from roots, as determined by the one-way ANOVA Dunnett’s test (*P < 0.05; **P < 0.01)

To explore the tissue expression patterns of *SmEXPs*, qRT-PCR was used to detect the expression levels of 18 *SmEXPs* members in four organs (roots, stems, leaves, and flowers) of *S. miltiorrhiza* (Fig. 5). The results showed that although *SmEXP* members were expressed in roots, stems, leaves, and flowers, their expression levels generally varied. The same genes had significant differences in their expressions in the four organs, and most *SmEXPs* had low expression levels in roots and high expression levels in flowers.

The *SmEXPA5* expression level was highest in the leaves and flowers, which indicated that it played important roles in their growth. The expression levels of *SmEXPA2* and *SmEXLA1* were highest in the root, implying their important roles in this organ.

### Analysis of *SmEXP* expression patterns under ABA treatments

**Fig. 6:**
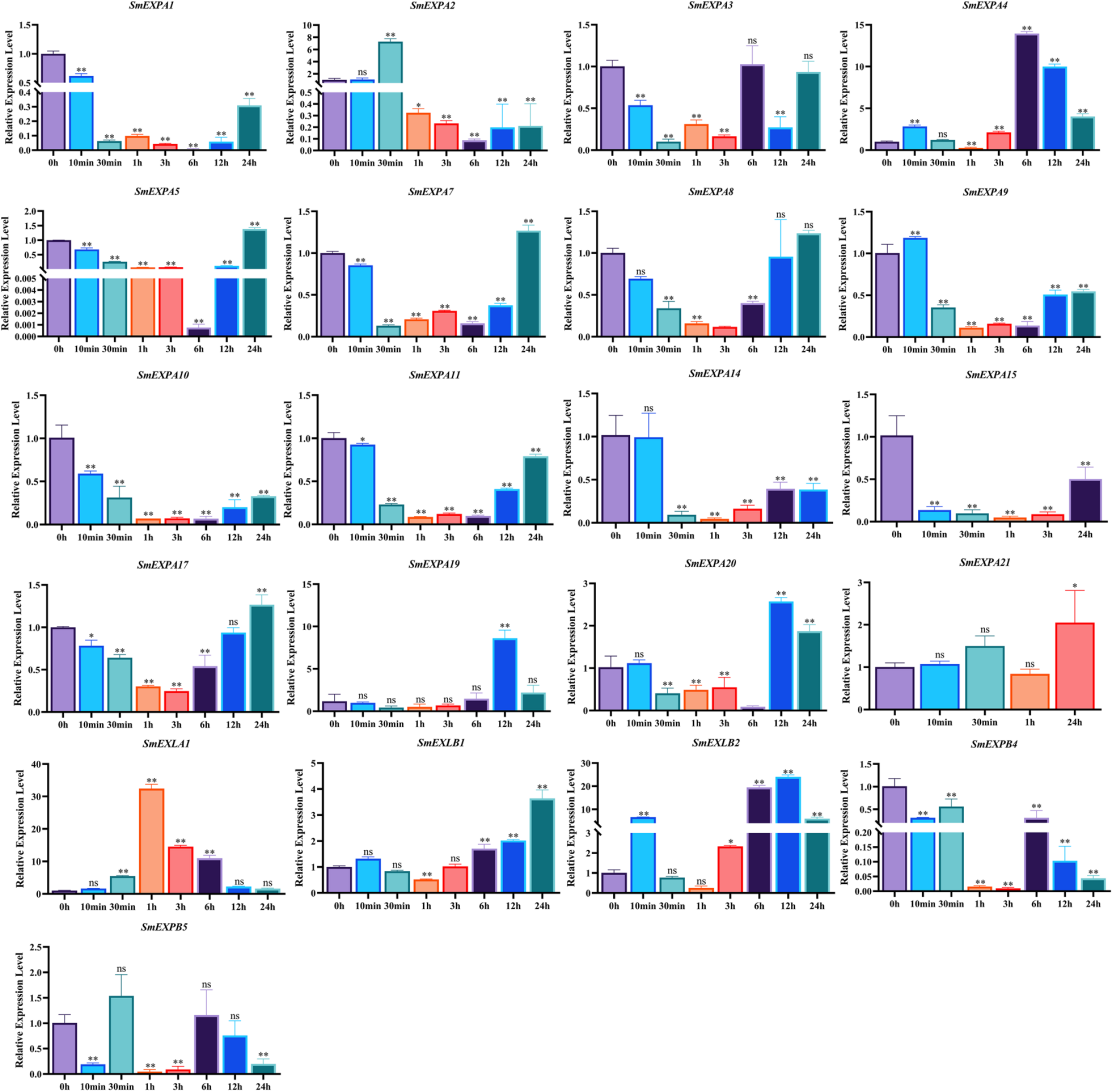
Expression of *SmEXP* treated with 100 μM ABA for 0 h, 10 min, 30 min, 1 h, 3 h, 6 h, 12 h, and 24 h, as determined by RT-qPCR. Data are shown as means ± SD, n = 3. Asterisks indicate significance differences from 0 min, as determined by a one-way ANOVA Dunnett’s test (*P < 0.05; **P < 0.01)

The analysis of *cis*-acting elements based on *SmEXP* promoters revealed that *SmEXP* members had a large number of ABA responsive elements. *S. miltiorrhiza* was treated with 100 μM of ABA and sampled at 0 h, 10 min, 30 min, 1 h, 3 h, 6 h, 12 h, and 24 h. The expression levels of 21 *SmEXPs* genes were detected by qRT-PCR (Fig. 6).

The results revealed that all 21 *SmEXPs* responded to ABA treatments, and ABA could negatively regulate the expressions of genes such as *SmEXPA1*, *SmEXPA3*, *SmEXPA5*, *SmEXPA7*, *SmEXPA8*, *SmEXPA10*, *SmEXPA11*, *SmEXPA14*, and *SmEXPB5*, etc. After 6 h of ABA treatment, the expression level of *SmEXPA5* decreased ~ 1341 times, while after 1 h of ABA treatment the expression level of *SmEXLA1* increased ~ 32 times. These results indicated that *SmEXPA5* and SmEXLA1 played a more important role in responding to ABA treatments in contrast to other *SmEXPs*.

### Analysis of *SmEXP* expression patterns under NaCl treatments

**Fig. 7:**
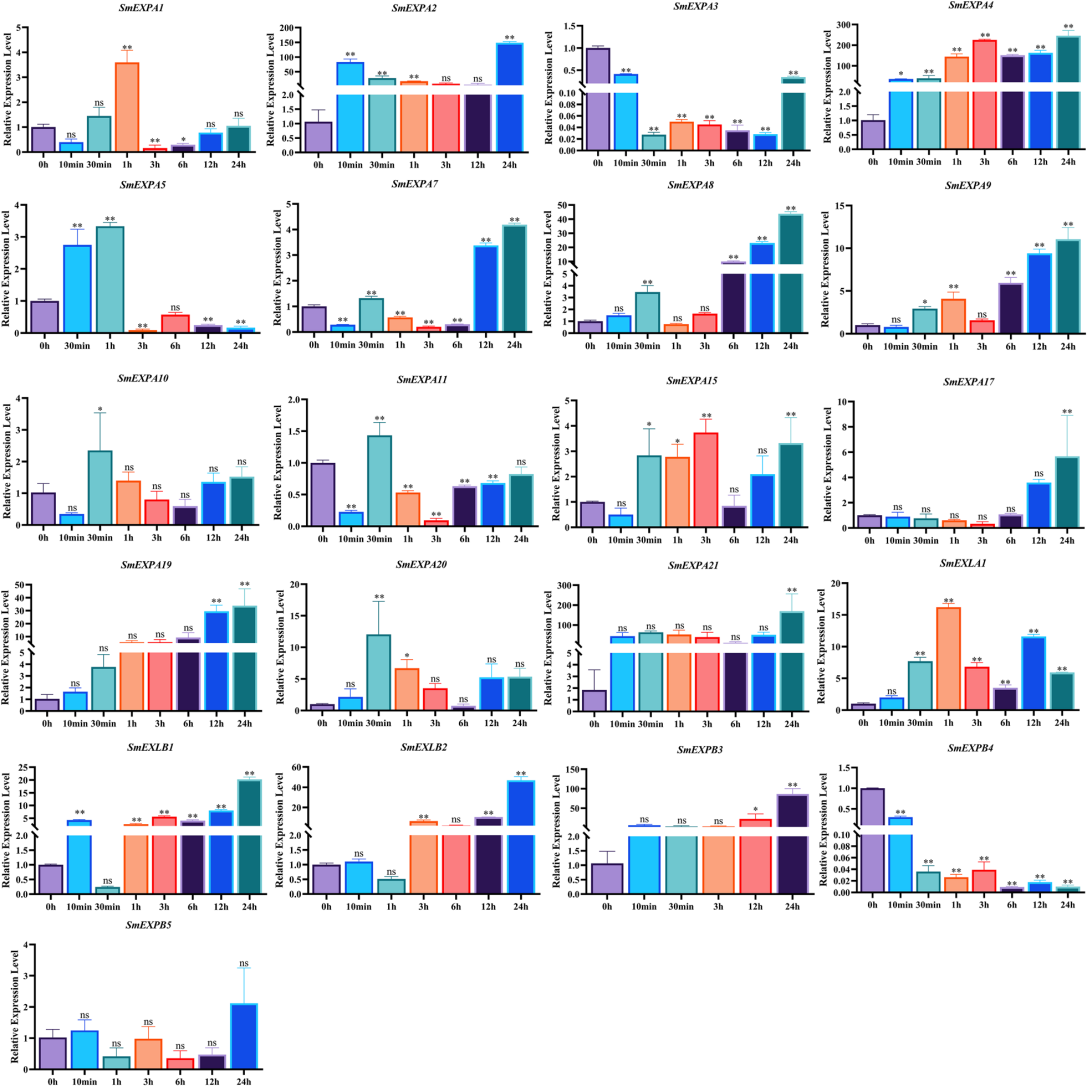
Expression levels of *SmEXP* treated with 100 mM NaCl for 0 h, 10 min, 1 h, 3 h, 6 h, 12 h, and 24 h, as determined by RT-qPCR. Data are shown as means ± SD, n = 3. Asterisks indicate significant differences from 0 h, as determined by one-way ANOVA Dunnett’s test (*P < 0.05; **P < 0.01)

After *S. miltiorrhiza* was treated with 100 mM NaCl for 0 h, 10 min, 1 h, 3 h, 6 h, 12 h, and 24 h, and the gene expression levels of 21 *SmEXPs* members were detected by qRT-PCR (Fig. 7). The results indicated that after the 100 mM NaCl treatment (except for *SmEXPB5*) the expression levels of the other *SmEXP* were not significantly altered, as were most of the upregulated genes. The expression levels of *SmEXPA2*, *SmEXPA4*, and *SmEXPA21* were upregulated ~ 149-fold, 246-fold, and 170-fold respectively at 24 h, whereas the expression level of *SmEXPA3* was downregulated by ~ 36-fold at 30 min. These results indicated that the *SmEXPA2*, *SmEXPA4*, *SmEXPA21*, and *SmEXPA3* played more important roles in the responses of the *SmEXP* family under salt stress.

### Analysis of *SmEXPs* expression patterns under Cu^2+^ treatments

**Fig. 8:**
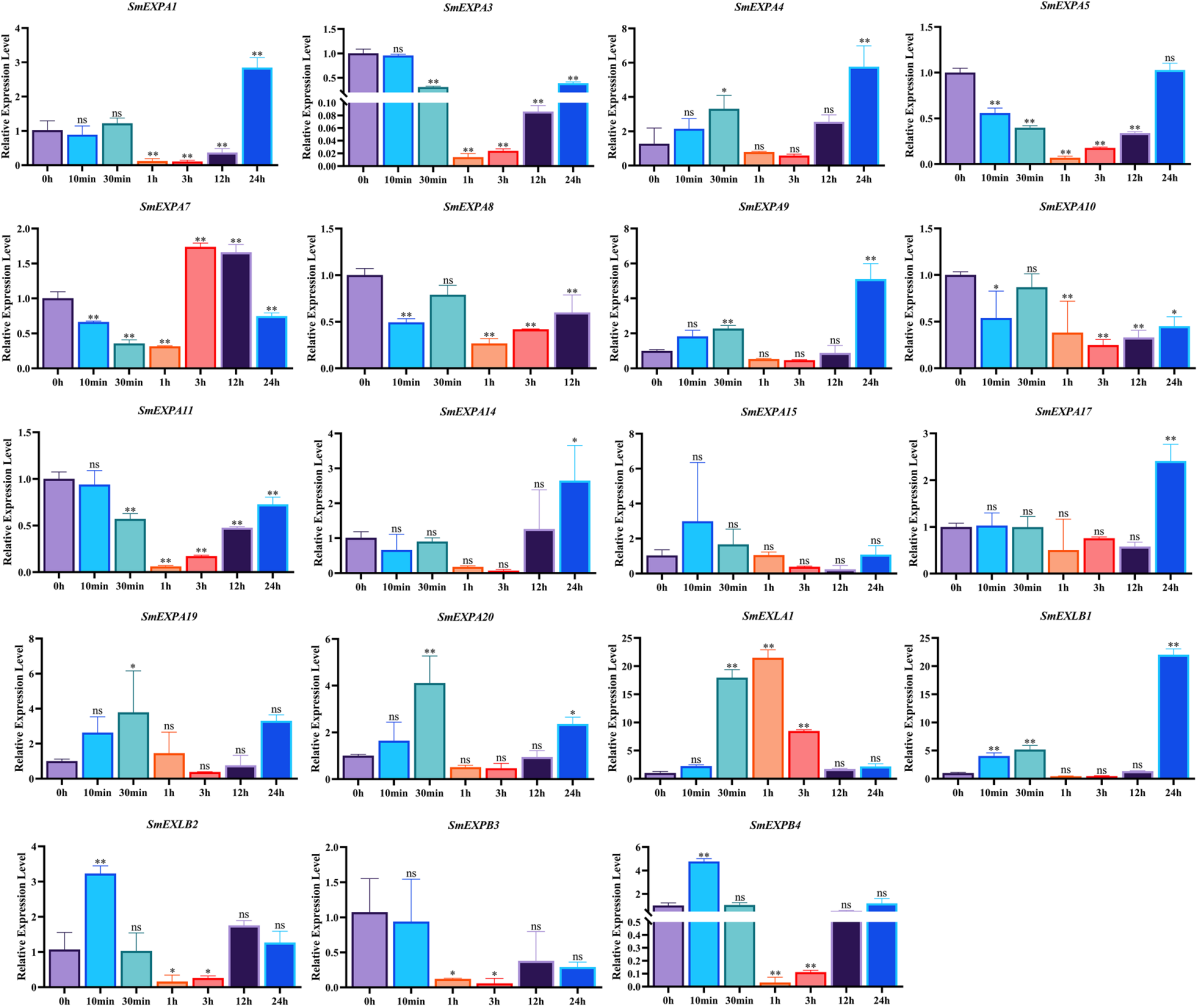
Expression of *SmEXP* treated with 200 μM Cu^2+^ for 0 h, 10 min, 30 min, 1 h, 3 h, 12 h, and 24 h. As determined by RT-qPCR. Data are shown as means ± SD, n = 3. An asterisk indicates significance differences from 0 h, as determined by one-way ANOVA Dunnett’s test (*P < 0.05; **P < 0.01)

Following the treatment of *S. miltiorrhiza* with 200 μM Cu^2+^ for 0 h, 10 min, 30 min, 1 h, 3 h, 12 h and 24 h, the gene expression levels of 19 *SmEXPs* members were detected by qRT-PCR (Fig. 8). Following the exogenous application of Cu^2+^ (except for *SmEXPA15*) the expression levels of most *SmEXPs* exhibited significant changes. The expression level of *SmEXPA3* decreased ~ 42 times after 1 h of treatment with Cu^2+^, while the expression level of *SmEXLB1* increased 22 times after 24 h of treatment. These results indicated that *SmEXPA3* and *SmEXLB1* played important roles in the *SmEXP* family when *S. miltiorrhiza* was contaminated with heavy metals.

### Subcellular localization of SmEXLA1, SmEXLB1, and SmEXPA2

**Fig. 9:**
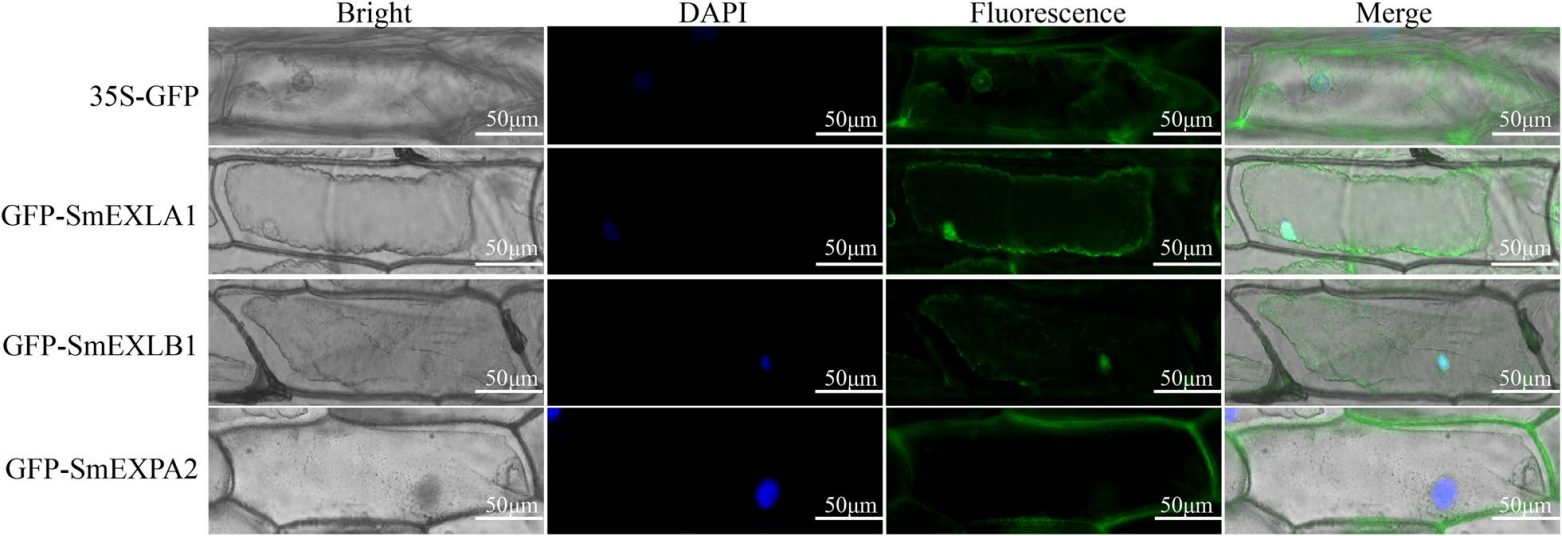
Subcellular localization analysis of GFP-SmEXLA1, GFP-SmEXLB1, and GFP-SmEXPA2. With 35S-GFP as a positive control, the localization of GFP-SmEXLA1, GFP-SmEXLB1, and GFP-SmEXPA2 in onion was confirmed by fluorescence microscopy (Scale bar = 50 μm)

To reveal the potential functions of SmEXLA1, SmEXLB1m, and SmEXPA2 we developed GFP-SmEXLA1, GFP-SmEXLB1, and GFP-SmEXPA2 protein expression vectors, and used 35-GFP vector as positive control. These vectors were then transferred into the inner epidermis of onion and the transient expression was observed under laser confocal microscopy. The positive control 35-GFP was expressed in the cytoplasm and nucleus, which was consistent with the biological state. The results confirmed that SmEXLA1 and SmEXLB1 were localized in the nucleus and cell membrane, and SmEXPA2 was localized in the cell wall (Fig. 9).

## Discussion

EXP is a protein that has the capacity to relax the cell walls of plants, which is extensively found in various plants and widely regarded as a key regulator of cell wall extension during the growth of plants [8]. In this study, SmEXP was identified and analyzed at the whole genome level. A total of 29 SmEXPs were identified based on the criteria containing both Pollen_allerg_1 and DPPB_1 domains [9].

Through phylogenetic analysis, SmEXPs were found to be distributed among four subfamilies, 21 SmEXPA, 5 SmEXPB, 1 SmEXLA, and 2 SmEXLB, which is similar to the subfamily proportions of most species. However, the number of SmEXP was generally lower than that of other plants [e.g., *A. thaliana* (35), *O. sativa* (58), *Moso Bamboo* (82) [54], *Solanum tuberosum* [55] (34), and *Zea mays* (88)] [56]. This indicated that repetitive events or the double amplification of *EXP* in the genome occurred less frequently during the evolutionary process of *S. miltiorrhiza,* which resulted in fewer members of the gene family.

Orthologous genes typically retain similar biological functions; thus, the identification of orthologous genes is very important in terms of the functional data of model plants to predict the functionalities of non-model plant genes [57]. Through a comparative investigation, 13 pairs of orthologous genes were selected from *SmEXP* and *AtEXP*, with a sequence consistency of > 50%. SmEXPA11 and AtEXPA4 were orthologous genes with a sequence consistency of 79.69%, where AtEXPA4 was observed to affect root growth [58]. The overexpression of *AtEXPA4* can enhance the elongation of primary roots, while knocking out *AtEXPA4* decelerates the growth rate of the primary root, which may also affect the root growth of *S. miltiorrhiza* in *SmEXPA11*.

AtEXPA18 is associated with root hair-specific 13 (RHS13), Peroxidase 7 (PER7), Proline-rich protein 3 (PRP3), Xyloglucan 14 (XTH14 endotransglucosylase/hydrolase protein), Pollen Ole e 1 allergen, extensin family protein (T30B22. 16), Probable 46 (PME46 pectinesterase/pectinesterase inhibitor), Peroxidase (PER73) 73, Putative pectinesterase/pectinesterase inhibitor 24 (PME24), Pollen Ole e 1 allergen, extensin family protein (MOP10), Leucine-rich repeat Extensin-like protein 10 genes (including 1 (LRX1)), which interacted with each other. These genes were associated with cell wall construction and root hair morphology. This prediction suggested that SmEXPA19 (a orthologous gene of AtEXPA18) may also play an important role in cell wall development and root hair growth.

An exploration of the expression patterns of *SmEXPs* in different tissues is of great significance for functional analysis. *SmEXPs* are expressed in the roots, stems, leaves, and flowers of *S. miltiorrhiza*, indicating that *SmEXPs* are broadly involved in its growth and development. Numerous ABA responsive elements were predicted for the *SmEXPs* promoter, and experiments revelaed that all 21 *SmEXPs* responded to ABA treatments.

The large accumulation of Na^+^ in soil can lead to the decreased water absorption capacities of plant roots, as well as damage to leaf cells through transpiration, which affects plant growth and, in severe cases, can lead to plant death [59–61]. When plant roots are subjected to salt stress, the earliest signals of this condition are received by the cell wall sensing system [62]. In response to salt stress, most *SmEXP* are activated, among which *SmEXPA3* and *SmEXPA4* have the strongest responses, and play the most important roles.

Under heavy metal stress, plants engage inherent mechanisms to reduce the absorption and accumulation of heavy metals. Excessive copper ions can alter the compositions and distribution of sugars in the cell walls of *Arabidopsis* roots, and copper can increase the contents of cellulose, hemicellulose, and pectin, which thicken cell walls to prevent harmful heavy metal ions from entering the cell [63]. It was found that Cu^2+^ can induce most *SmEXP* expression; however, the kinetics that drive the exogenous Cu^2+^ induction of *SmEXP* differential expressions require further in depth investigation.

Subcellular localization prediction results revealed that all *SmEXPs* were located on the cell wall. It was verified through subsequent experiments that *SmEXPA2* was indeed located on the cell wall and belonged to its complement of proteins. However, SmEXLA1 and SmEXLB1 were not present on the cell wall as predicted, but on the nuclear membrane. Due to the diversity of EXPANSIN gene functions, all candidate *SmEXPs* will require further analysis to verify their functions during the growth and development of *S. miltiorrhiza*.

## Conclusion

For this study, 29 SmEXPs of *S. miltiorrhiza* were identified and analyzed at the whole genome level. They were analyzed from the aspects of systematic evolution, gene structure, protein characteristics, promoter analysis, tissue expression patterns, and cell localization. This revealed the basic characteristics of the SmEXP family and predicted their potential functions in biological processes, which lays a foundation for further research on specific functions. Further, we investigated the responses of SmEXP family members under various ABA and stress treatments, which is of great significance for the breeding and large scale production of *S. miltiorrhiza*.

### Supplementary Information

**Additional file 1: Table S1. **Primer sequences of qRT-PCR.

**Additional file 2: **Sequence of each SmEXP.

**Additional file 3: Fig.S1.** Full length amino acid sequence alignments of orthologous genes.

## Data Availability

The data analyzed during this study may be obtained from the corresponding author upon reasonable request.
