# Supplementary figures and images for: Identification and expression analysis of expansin gene family in Salvia miltiorrhiza
Source: Chin Med. 2024 Feb 4;19:22. doi: 10.1186/s13020-023-00867-w (PMC10838462; doi:10.1186/s13020-023-00867-w)

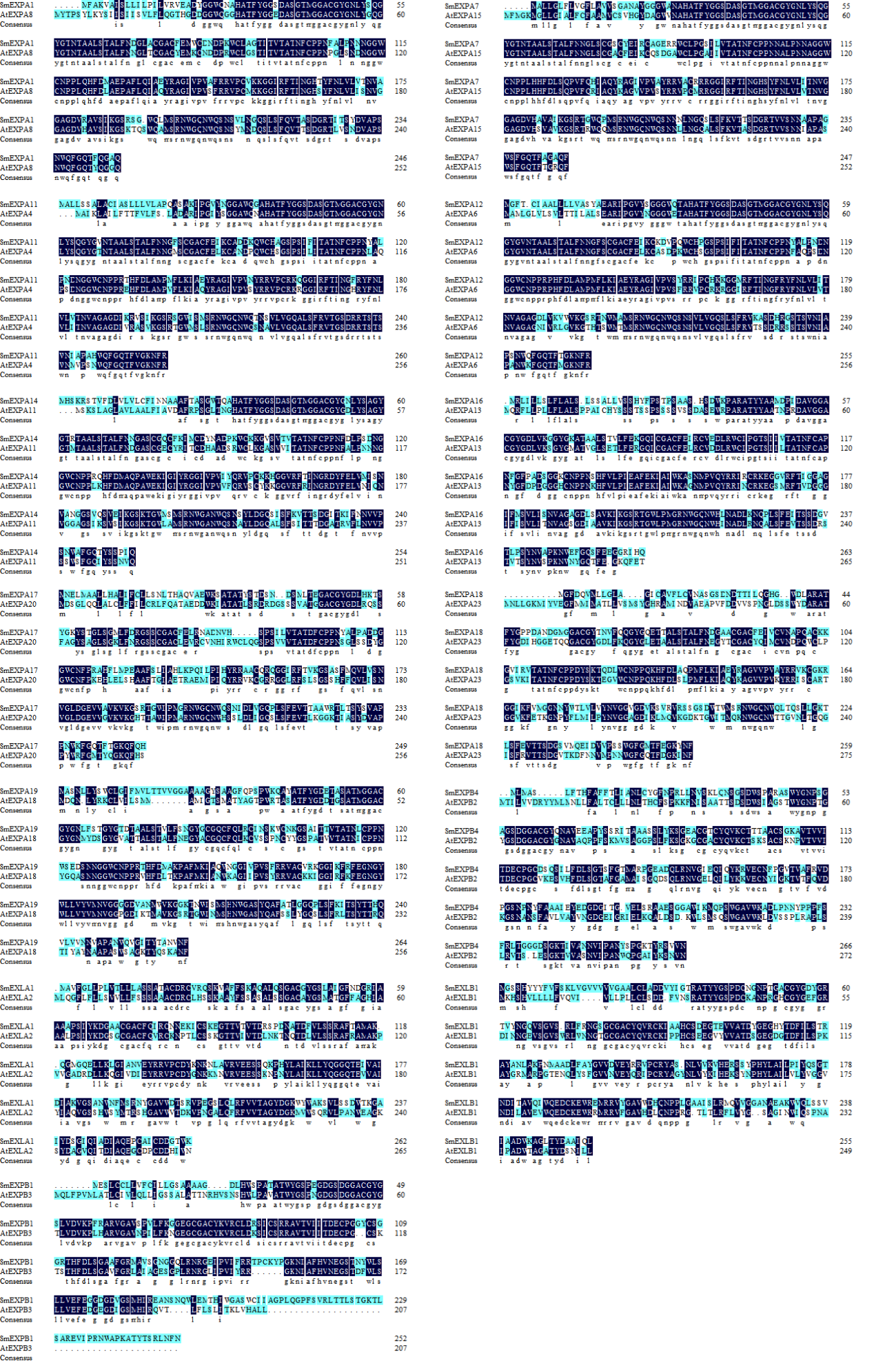


Fig.S1 Full length amino acid sequence alignment of orthologous gene.

Supplement: Supplementary file 3 — Additional file 3: Fig.S1. Full length amino acid sequence alignments of orthologous genes. [file 13020_2023_867_MOESM3_ESM.docx]
